# Scalable Microfabrication of Multi-Emitter Arrays in Silicon for a Compact Microfluidic Electrospray Propulsion System

**DOI:** 10.1021/acsami.2c12716

**Published:** 2022-09-16

**Authors:** Albert Cisquella-Serra, Marc Galobardes-Esteban, Manuel Gamero-Castaño

**Affiliations:** Department of Mechanical and Aerospace Engineering, University of California, Irvine, California 92617, United States

**Keywords:** electrospray, electric propulsion, MEMS, microfluidics, SmallSats, electrospray propulsion

## Abstract

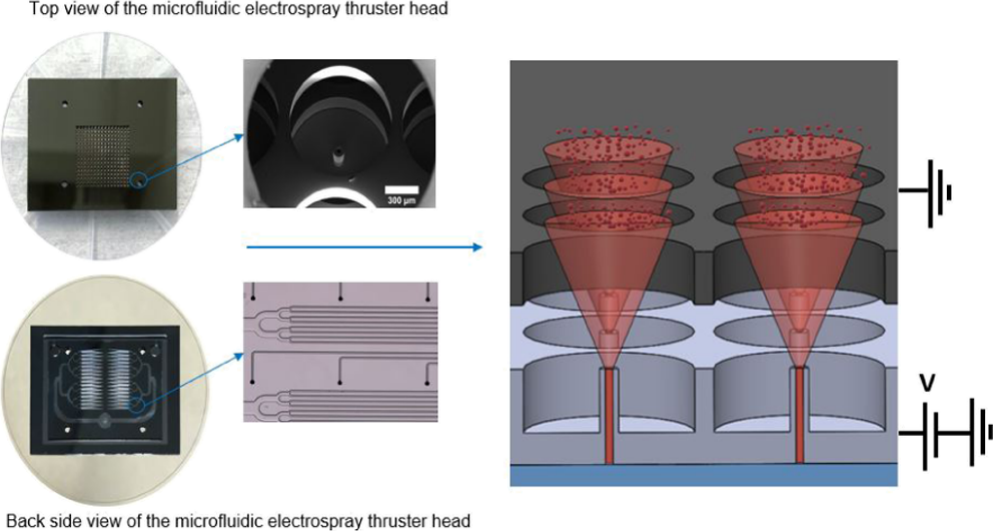

The recent proliferation of SmallSats and their use in increasingly demanding applications require the development of onboard electric propulsion compatible with the power, mass, and volume constraints of these spacecraft. Electrospray propulsion is a promising technology for SmallSats due to its unique high efficiency and scalability across the wide power range of these platforms, for example, from a few watts available in a CubeSat to a few hundred watts in a MiniSat. The implementation of electrospray propulsion requires the use of microfabrication techniques to create compact arrays of thousands of electrospray emitters. This article demonstrates the microfabrication of multi-emitter electrospray sources of a scalable size for electrospray propulsion. In particular, a microfabrication and assembly process is developed and demonstrated by fabricating sources with arrays of 1, 64, and 256 emitters. The electrospray sources are tested in a relevant environment for space propulsion (inside a vacuum chamber), exhibiting excellent propulsive performance (e.g., absence of beam impingement in the extractor electrode, absence of hysteresis in the beam current versus propellant flow rate characteristic, proper operation in the cone-jet electrospraying mode, etc.) and nearly coincident output per emitter. Several design elements contribute to this performance: the even distribution of the propellant among all emitters made possible by the implementation of a network of microfluidic channels in the backside of the emitter array; the small dead volume of the network of microfluidic channels; the accurate alignment between the emitters and extractor orifices; and the use of a pipe-flow configuration to drive the propellant through closed conduits, which protects the propellant.

## 1 Introduction

Electrospraying is a useful tool for atomizing liquids into charged droplets with controllable diameters as small as a few nanometers,^1^ and to desorb molecular ions from a liquid into the gas phase.^2^ Typically, the liquid is fed to the tip of an emitter electrode at high potential. The interplay between the electrostatic stress, the surface tension, and the flow shape the liquid into a so-called Taylor cone, from whose vertex a steady jet develops. At some distance from the vertex, the jet becomes unstable and breaks into charged droplets. The radii of the jet and charged droplets depend on the physical properties of the liquid and its flow rate. Ion field emission from the surface of a liquid requires electric fields of the order of 1 V/nm, which are only possible from surfaces with radii of curvature of a few nanometers.^3^ Electrosprays of highly conducting liquids naturally produce such surfaces.^4^ The ability to produce ions and charged droplets of controllable diameters down to a few nanometers has important technological applications in nanoparticle generation and deposition,^5−7^ mass spectrometry,^2,8^ sputtering,^9,10^ electrospinning of nanofibers,^11,12^ electric propulsion for spacecrafts,^13,14^ and so forth.

Electrosprays were recognized as useful for spacecraft propulsion in the 1960s.^15^ Interest resurfaced in the late 1990s due to the progress in miniaturization techniques that made it possible to scale down spacecraft subsystems, giving rise to the SmallSat category (spacecraft with masses under 600 kg). The lower costs associated with SmallSats, the decreasing service price of commercial rocket launchers, and the standardization of SmallSat platforms such as the CubeSat^16,17^ have boosted space activity in this sector in the last few years.^18^ For example, 1743 SmallSats were launched in 2021, compared to the 389 SmallSats put in orbit in 2019 and 39 in 2011, with 94% of the spacecraft launched in 2021 being SmallSats.^19^ On-board propulsion is an enabling capability for many SmallSat applications such as the deployment and maintenance of constellations; orbit insertion; orbit maintenance for increasing the mission life of individual satellites; and deorbit, which is likely to be required. Electric propulsion is an attractive option compared to chemical propulsion due to its larger specific impulse and the associated savings in propellant mass. However, traditional electric propulsion technologies such as Hall thruster and ion engines relying on a plasma discharge are ill-suited for the scale-down in size and power required by SmallSats, especially for microsats (<200 kg) and smaller platforms.^20,21^ Most CubeSats in orbit lack a propulsion system due to the complexity of scaling it down to the available volume, mass, and power budgets.^22,23^

Electrospray propulsion is based on the electrostatic acceleration of the charged droplets and ions emitted by an electrospray. A single emitter typically operates at the micronewton and milliwatt thrust and power levels, with a propulsive efficiency exceeding 70%.^13^ Arrays of thousands of emitters are fabricated with micromachining techniques to process the much higher power available for propulsion. The high efficiency across the power range of the SmallSat class (from a few watts in a CubeSat to a few hundred watts in a MiniSat), and the size scalability provided by microfabrication make electrospray propulsion an excellent option for SmallSats, both for primary propulsion and attitude control. Successful miniaturization of electrospray emitter arrays requires expertise in both MEMS fabrication and electrospray physics. There have been several attempts at miniaturizing actively fed emitter arrays, that is, designs in which an imposed pressure difference drives the propellant in a closed-conduit flow;^24−26^ these emitter arrays operate in either the droplet or the mixed droplet-ion emission modes. It is difficult to implement the high and well-matched hydraulic resistances needed to evenly distribute the propellant among all emitters. Suzuki et al. increased the hydraulic resistance by using submicrometer SiN capillaries. Recent attempts using novel technologies such as 3D microlithography and two photon-lithography to make emitters with highly restrictive channels have opened a new path for manufacturing capillary emitters but are still unable to deliver a steady emission.^27,28^ Passively fed emitter arrays have been an alternative and more successful approach. Typically made of porous materials, the flow of propellant in these emitters is driven by capillarity and the small suction pressure at the emitter tip generated by the applied potential.^29−31^ However, these thrusters report a loss of propulsion efficiency over time due to non-uniformities and large pores introduced by the randomness of the microfabrication process, which leads to hot spots in the emission current and shorting between emitters and the extractor.^32,33^ Actively fed emitter arrays operating in the droplet mode present advantages such as the protection of the propellant provided by closed conduits, as well as the continuous elimination of neutralized counterions which flow out of the system with the atomized propellant. Recently, Grustan-Gutierrez and Gamero-Castaño^34^ implemented adequate hydraulic resistance in an actively fed thruster by detaching it from the geometry of the emitter and instead placing it in a network of microfluidic channels. This article uses this approach to demonstrate a scalable microfabrication and assembly process for a compact silicon-based multi-emitter electrospray source. The sources are tested with the ionic liquid 1-ethyl-3-methylimidazolium bis(trifluoromethylsulfonyl) imide (EMI-Im or [EMIM^+^][NTf_2_^–^]),^35^ exhibiting excellent performance for propulsion. The electrospray sources demonstrated in this article could also be used for other applications such as nanoparticle deposition, electrospinning, and electrospray ionization.

## 2 Design and Fabrication

### 2.1 Basic Design of the Electrospray Source

**Figure 1:**
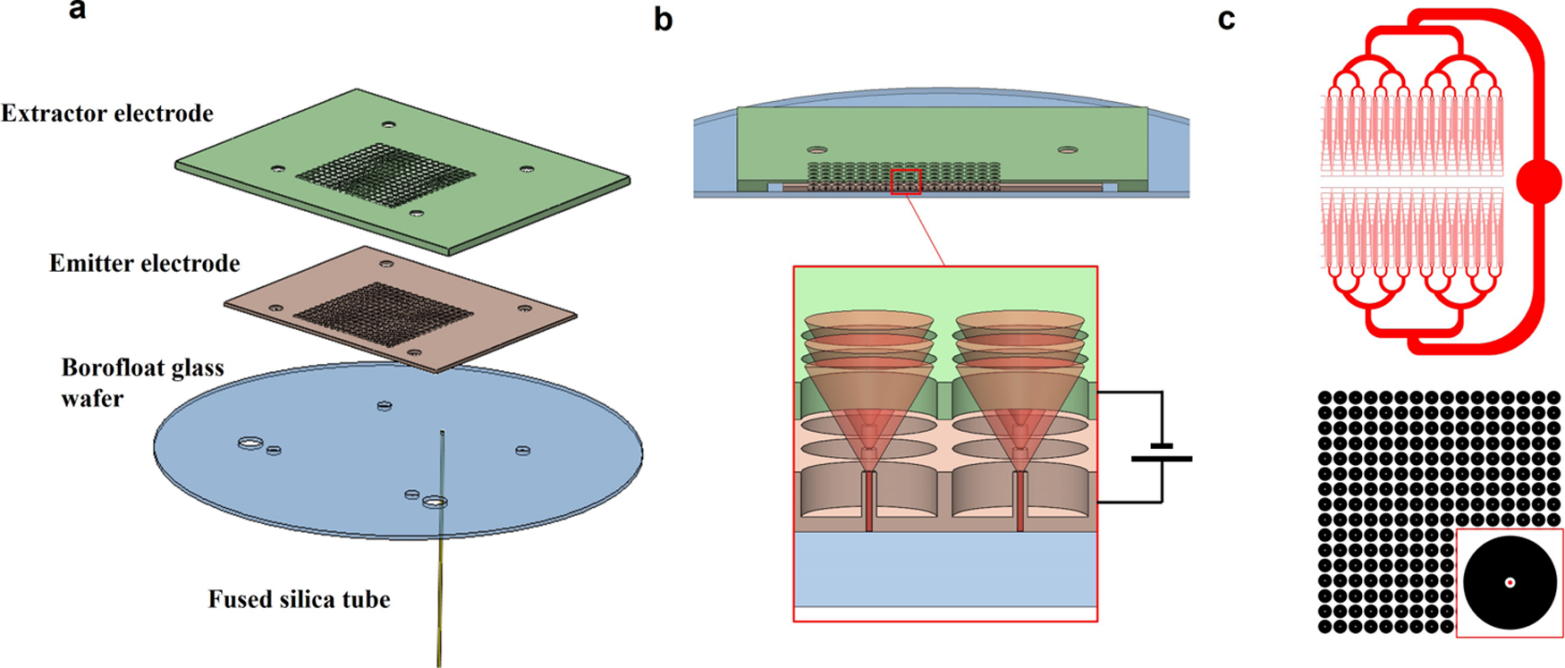
(a) Exploded view of the components of the electrospray source; (b) section view of the assembled electrospray source; and (c) backside and topside of the emitter electrode showing the network of microfluidic channels and the emitter array respectively.

The electrospray source includes the three elements shown in Figure 1a: an emitter and extractor electrodes micromachined in silicon, and a borosilicate glass wafer. The propellant is fed to the tips of cylindrical emitters through axial conduits and electrosprayed into charged droplets and ions by applying a voltage difference between the two electrodes (see Figure 1b). The voltage difference also accelerates the charged particles, exiting the source at high velocity through orifices in the extractor. Each emitter faces an orifice, and this pair is repeated to form a square array with 2^*N*^ elements. Because of the electrostatic shielding provided by the extractor, the electric field at the tip of each emitter is not affected by nearby emitters, nor by the space charge resulting from the superposition of beams downstream of the extractor, that is, the emitter-orifice pairs are electrostatically decoupled. Because the hydraulic resistance that can be implemented in the circular conduit of each emitter is insufficient to divide the flow rate of the propellant evenly among them, a larger flow restriction is imposed using microfluidic channels etched on the back side of the silicon wafer (see Figure 1c). A fused silica tube feeds the propellant to a circular pool from which the flow is bifurcated *N* times. Each bifurcation creates a pair of identical channels with half of the cross section of the parent channel. Each one of the 2^*N*^ final channels discharges into the axial conduit of an emitter, and their width and length make them the major flow restrictors. Because the width and depth of these 2^*N*^ channels can be etched with small tolerances, and because they are the main contributors to the hydraulic resistance, the network of channels evenly distributes the propellant among the emitters. The backsides of the emitter and the extractor are anodically bonded to the glass wafer while ensuring proper alignment using precision rods, which are inserted through matching patterns of holes etched in the three wafers. The glass wafer also provides electrical insulation between the electrodes and seals the open side of the microfluidic channels.

### 2.2 Emitter Electrode

**Figure 2:**
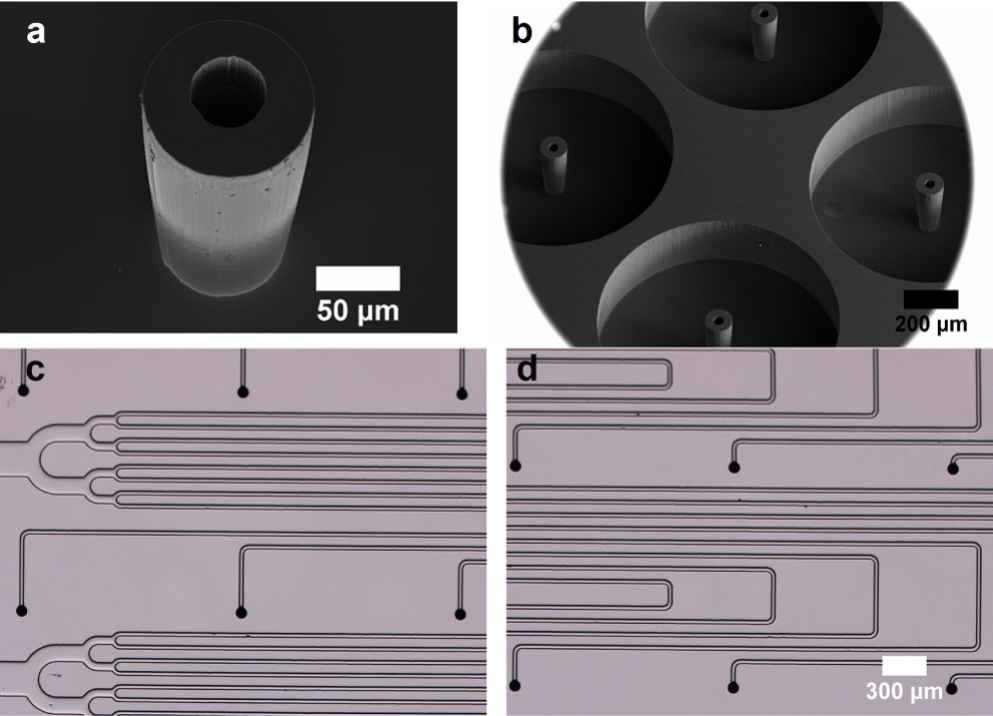
(a,b) SEM pictures of emitters and surrounding wells (the stage is tilted 20°); (c,d) branching of the network of microfluidic channels and transition with emitter conduits.

The emitter electrode is made with a double-side polished silicon wafer patterned with a square array of emitters on the topside, and a network of microfluidic channels on the backside. Figure 2a,b shows scanning electron microscopy (SEM) images of a single emitter and of a group of four emitters.

**Figure 3:**
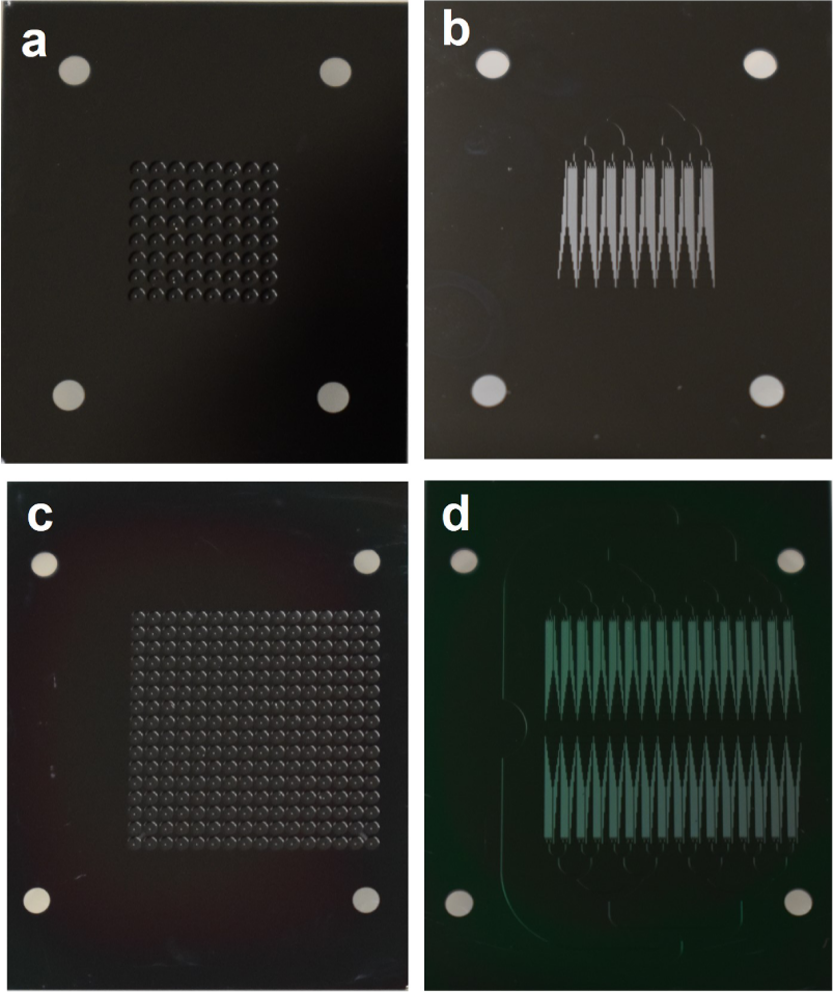
(a) Topside of a 64 emitter array electrode; (b) backside of a 64 emitter array electrode showing the network of microfluidic channels; (c) topside of a 256 emitter array electrode; and (d) backside of a 256 emitter array electrode.

Each emitter is a cylindrical tube formed by etching a surrounding well and an axial, circular conduit. The etching of the well determines the height of the emitter, while the conduit is etched through the silicon wafer and connects with a microfluidic channel. The height, outer diameter, and inner diameter of the emitters are 275, 100, and 40 μm, respectively; the diameter of the well is 900 μm, and the pitch between emitters is 1 mm. Figure 2c,d shows photographs of the microfluidic channel network, showing the successive branching of the channels as well as the transition between the ends of the final channels and the conduits of the emitters (seen in the photographs as black circles). Figure 3a shows a photograph of the topside of a 64 emitter electrode, which has an area of 2.6 × 2.2 cm^2^. Note the 8 × 8 square array of emitters, and the four holes with diameters of 1.65 mm used for the alignment. Figure 3b is a photograph of the backside of the 64 emitter array electrode showing the network of microfluidic channels. The single main channel starting the network has a width of 620 μm, while the final 64 channels feeding the emitters have widths of 20 μm and identical lengths of 7500 μm. All channels in the network have the same nominal depth, which is controlled with a calibrated inductively coupled plasma reactive ion etching (ICP-RIE) process. Figure 3c,d shows the topside and backside of a 256 emitter electrode, which has an area of 3.4 × 2.6 cm^2^. The microfluidic channel network repeats four times the network of the 64 emitter array, and has two additional branching levels for connecting to a single propellant intake.

The fabrication of the emitter array electrode starts with a RCA cleaning and dehydration of the silicon wafer. This step is repeated after each lithography and etching during the fabrication. The pattern with the microfluidic channels and alignment holes is transferred to the back side of the wafer by photolithography using an AZ positive photoresist. The microfluidic channels and alignment holes are then etched using ICP-RIE with a modified Bosh Recipe. An etch-rate calibration is used to stop the etching of the microchannels at the depth needed for the desired hydraulic resistance. The photoresist is stripped, and a 1 μm layer of SiO_2_ is grown using dry thermal oxidation. This layer is used for both protecting the microfluidic channels and as a stopping layer for the last etching step. A 3 μm layer of SiO_2_ is deposited on the topside of the wafer using plasma-enhanced chemical vapor deposition (PEVCD). The emitter array pattern is then transferred on top of the deposited SiO_2_ layer using photolithography with an AZ positive resist. Backside alignment is used to match the center hole of the emitters with the end of the microfluidic channels. The emitter pattern is etched on the 3 μm layer of SiO_2_ using RIE with a SiO_2_ etch recipe. The photoresist is stripped and another lithography is performed exposing only the inner channel of the emitters and the alignment holes. ICP-RIE with a modified Bosch recipe is used to etch both the inner channel of the emitters and the alignment holes. While the latter are etched through the wafer, the inner channels are etched only 60 to 70% of the wafer thickness. The photoresist is stripped, exposing the SiO_2_ mask with the geometry of the well surrounding the emitter and its inner channel. A final ICP-RIE process finishes the inner channels and etches the wells surrounding the emitters down to a depth of 275 μm. Finally, the SiO_2_ is removed from the emitter array electrode by soaking it in a buffered oxide etch bath (BOE 6:1) for 1 h.

### 2.3 Extractor Electrode

**Figure 4:**
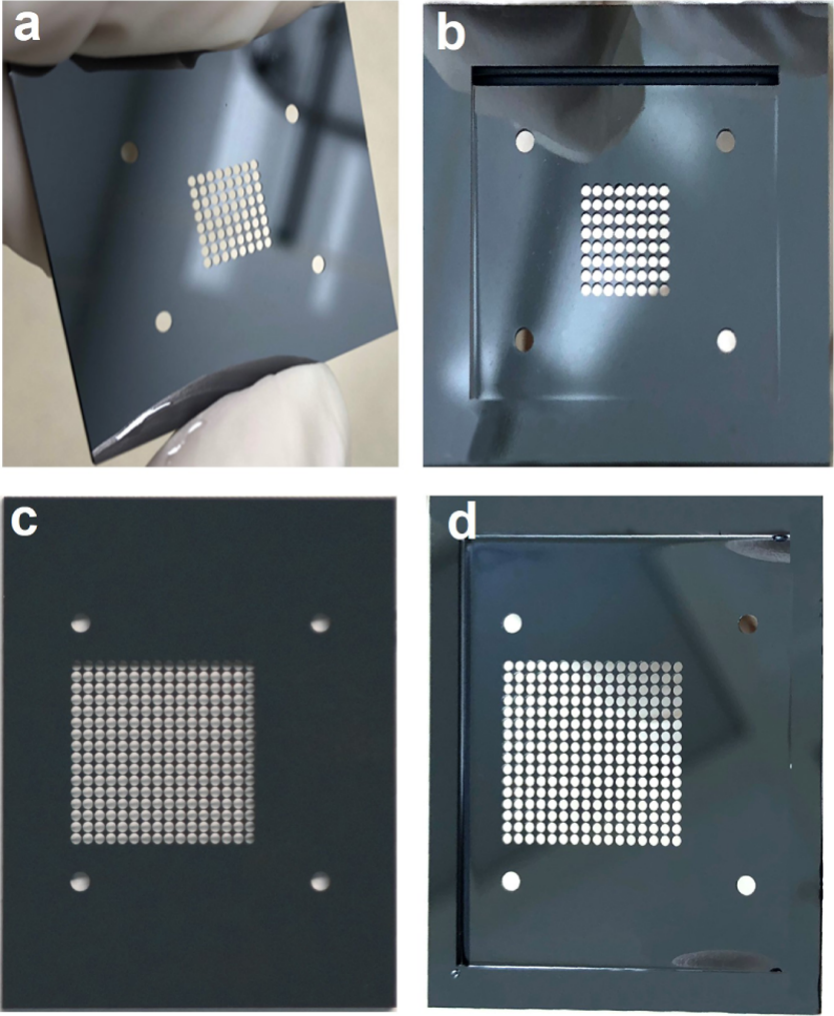
(a) Topside of the extractor electrode for the 64 emitter source; (b) backside of the extractor electrode for the 64 emitter source; (c) topside of the extractor for the 256 emitter source; and (d) backside of the extractor for the 256 emitter source.

The extractor electrode is made with a double-side polished silicon wafer 1 mm thick. Figure 4a,b shows the topside and backside of the extractor electrode for the 64 emitter array. A square array of extractor orifices, matching the square array of emitters, is etched through to allow the passage of the electrospray beamlets. Each orifice has a diameter of 0.9 mm. Note the four large orifices (1.65 mm in diameter) used for alignment. The topside of the extractor is a flat 3.2 × 3.6 cm rectangle, while the backside has a 2.32 × 2.72 cm rectangular trench. This trench encases the emitter electrode once the source is assembled, while providing a gap between both electrodes for electrical isolation. Figure 4c,d shows the extractor electrode of the 256 array source, with 3.40 × 4.43 cm sides and a 2.9 × 3.7 cm trench. The depth of the trench is controlled with a calibrated time-stop ICP-RIE etch, and is always larger than the thickness of the emitter electrode.

The fabrication of the extractor electrode also starts with a RCA cleaning and dehydration of the silicon wafer, which are repeated after each lithography and etching. The pattern with the square array of extraction orifices and the four alignment holes is transferred on the topside using photolithography with an AZ positive photoresist. The pattern is then etched using ICP-RIE with a modified standard Bosch recipe. The photoresist is stripped, and a 4 μm layer of SiO_2_ is deposited on the backside using PEVCD. A mask of AZ positive photoresist is patterned on top of the SiO_2_ layer to define the large rectangular area needed to etch the trench that will encase the emitter electrode. The SiO_2_ is etched using RIE, creating a SiO_2_ mask for the deep etch of the backside of the silicon wafer. The exposed silicon is etched with ICP-RIE using a slow Bosch process (the etching area and depth are significant) to guarantee a uniform profile in the face of the extractor facing the emitters. The SiO_2_ is then removed with a BOE bath.

### 2.4 Micromachined BOROFLOAT Glass and Anodic Bonding of the Components

**Figure 5:**
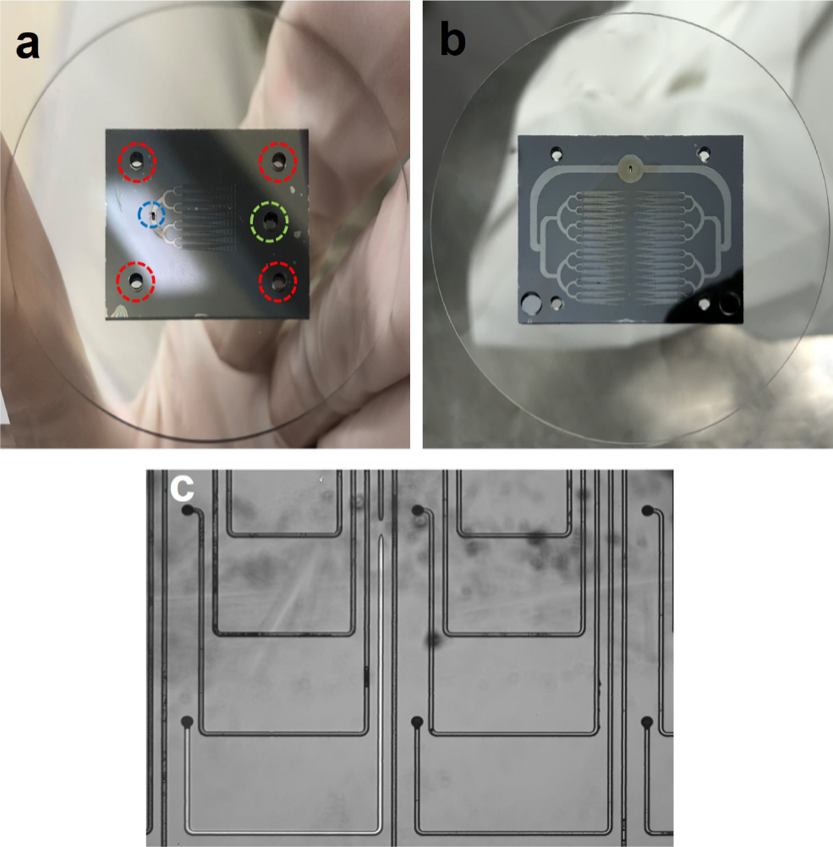
(a) Back side view of the 64 emitter electrode bonded to the glass wafer, with colored circumferences pointing to the location of orifices in the glass wafer; (b) similar back side view of the 256 emitter source; and (c) test showing the absence of propellant leaks in the sealed network of microfluidic channels.

The network of microfluidic channels is sealed with a BOROFLOAT glass wafer anodically bonded to the backside of the emitter electrode. The backside of the extractor electrode is also anodically bonded to the glass wafer, encasing the emitter electrode and permanently integrating the three elements of the electrospray source. Four precision zirconia rods are inserted through the 1.65 mm orifices during bonding to accurately align the emitter and the extractor arrays. Figure 5a,b shows the glass wafers bonded to the 64 and 256 emitter electrodes before bonding the extractors; their diameters are 50 and 60 mm, respectively. The glass wafers have several through-holes made with femtosecond laser induced selective etching: a small orifice with a diameter of 370 μm for inserting the fused silica line that feeds the propellant (a blue circumference marks its position in Figure 5a); the pattern of four alignment holes (red circumferences); and an orifice to access the emitter electrode and connect it to a high voltage power supply (green circumference). We routinely inspect the backside of the emitter electrode with a microscope after operating the electrospray source to verify that the glass wafer and the anodic bonding properly seal the microfluidic channels. We have never observed propellant leaking out of any channel. Figure 5c shows an extreme test case in which the etching of one channel was purposely discontinued. The photograph was taken after filling the source with propellant and operating it inside a vacuum chamber (see Section 4). When the propellant is fed to the emitters, the fluid in the discontinued channel is at the highest pressure in the feed system; however, no propellant leaks out. Furthermore, no transfer of propellant occurs from the channels with pressurized propellant to the final section of the discontinued channel (this channel, being empty, appears lighter).

**Figure 6:**
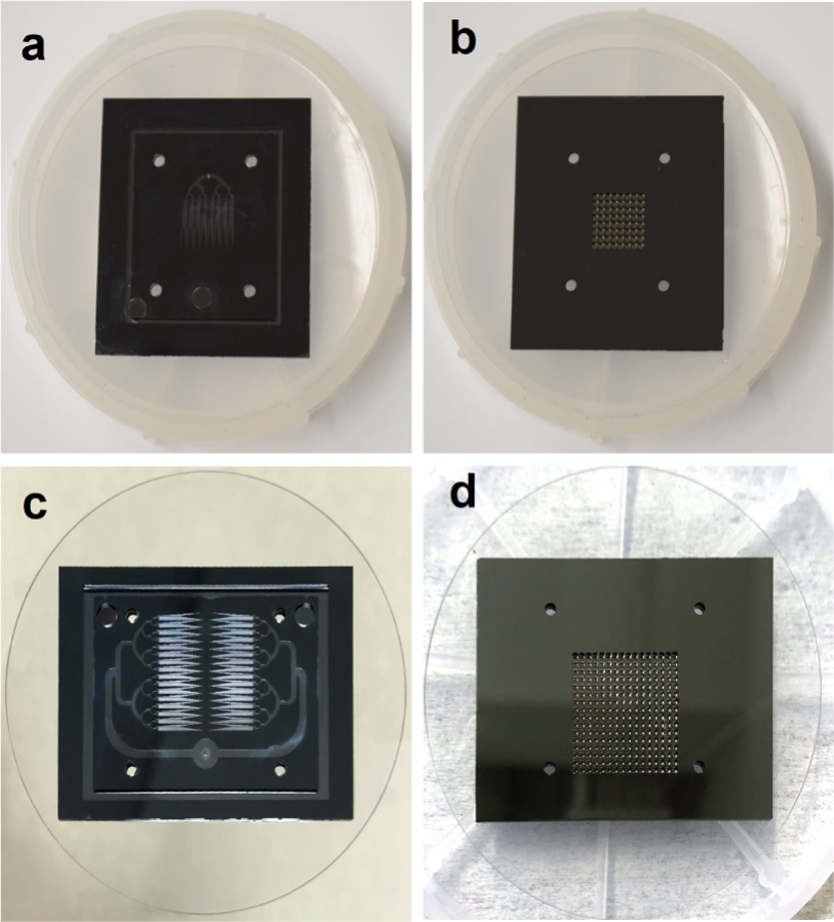
(a) Back side view of the 64 emitter source (after bonding the emitter, extractor, and glass wafers); (b) top side view of the 64 emitter source; (c) back side view of the 256 emitter source; and (d) top side view of the 256 emitter source.

Figure 6a,b shows bottom and top views of the three elements bonded into a 64 emitter source. The bottom view displays the gap between the electrodes, as well as the network of microfluidic channels. The vacuum gap and the electrical insulation provided by the glass substrate make it possible to apply a voltage difference of several kilovolts between the emitter and extractor electrodes without current leakage, which is needed to electrospray the propellant. Figure 6c,d shows top and bottom views of the three elements bonded into a 256 emitter source.

**Figure 7:**
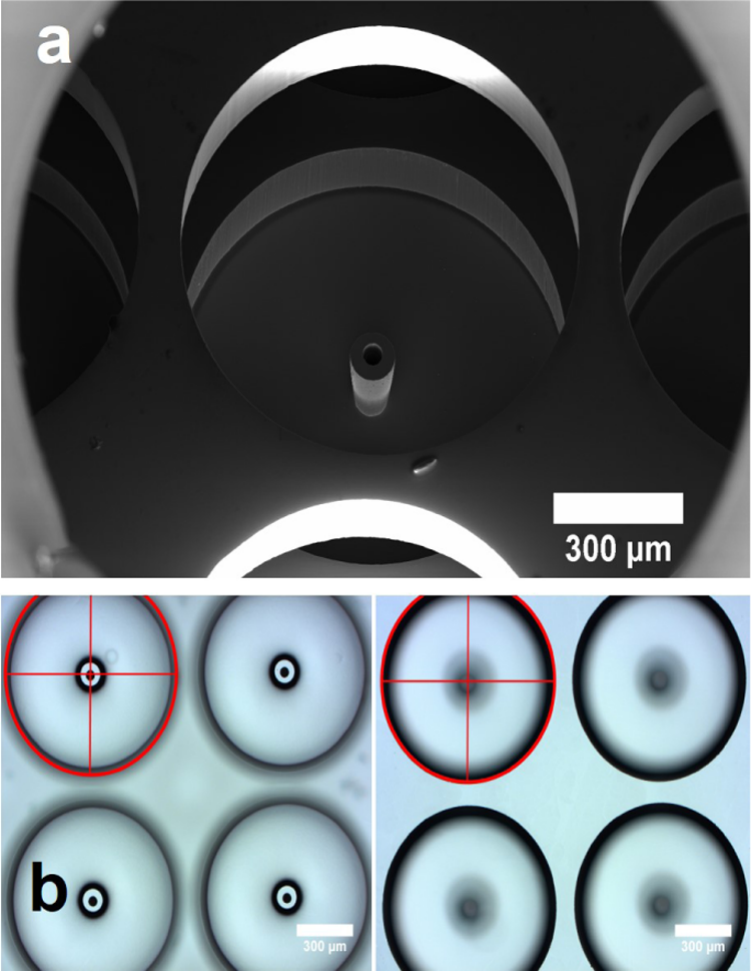
(a) SEM image of the emitter/extractor region for one emitter; (b) photographs focused on emitter tips and extractor rims showing their alignment.

Figure 7a is an SEM image of an emitter and its surrounding well, its extractor orifice and the gap between them. Figure 7b illustrates the alignment with photographs focused on either the tips of the emitters or on the rims of the extractor orifices. We have also fabricated and tested a single emitter source using the same fabrication and assembly steps. All sources have excellent voltage isolation and alignment between the extractor orifices and the emitters.

## 3 Experimental Setup and Methods

**Figure 8:**
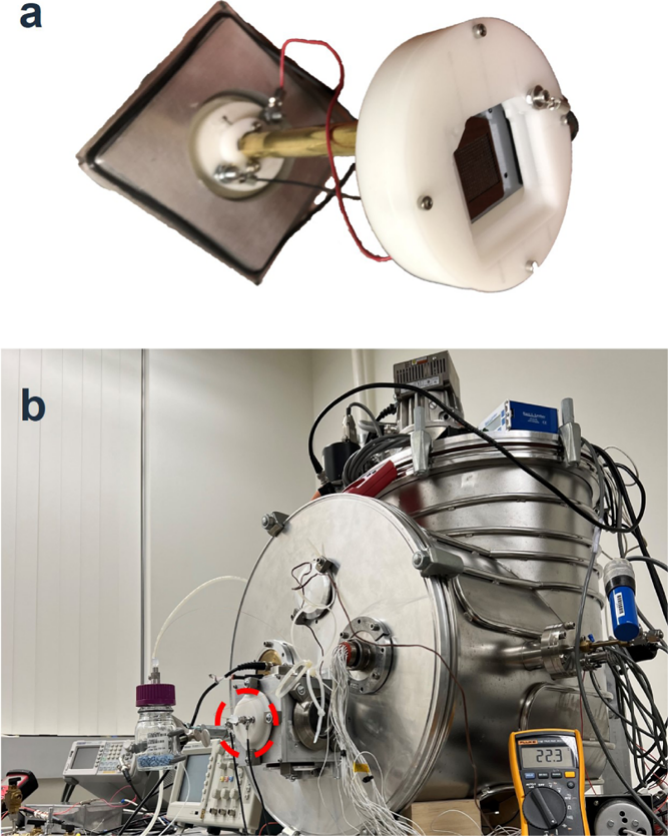
(a) 256 emitter electrospray source mounted on a vacuum flange; (b) vacuum chamber used for the testing the electrospray source.

Figure 8a shows a Delrin fixture holding a 256 emitter source and mounted on a vacuum flange for testing. The electric contacts for the emitter and extractor electrodes are made with metallic springs compressed by screws threaded in the Delrin fixture, to which copper leads are attached. The fused silica line attached to the electrospray source and feeding the propellant is passed through the vacuum flange using an Upchurch UPF-120 finger-tight fitting. The flange is mounted on the stainless-steel vacuum chamber shown in Figure 8b, which is served by a Varian TV-301 turbo molecular pump backed by a Sargent Welch DuoSeal 1397 mechanical pump. During testing the background pressure, measured with a Kurt J. Lesker 423 cold cathode gauge, is below 5 × 10^–6^ Torr.

**Figure 9:**
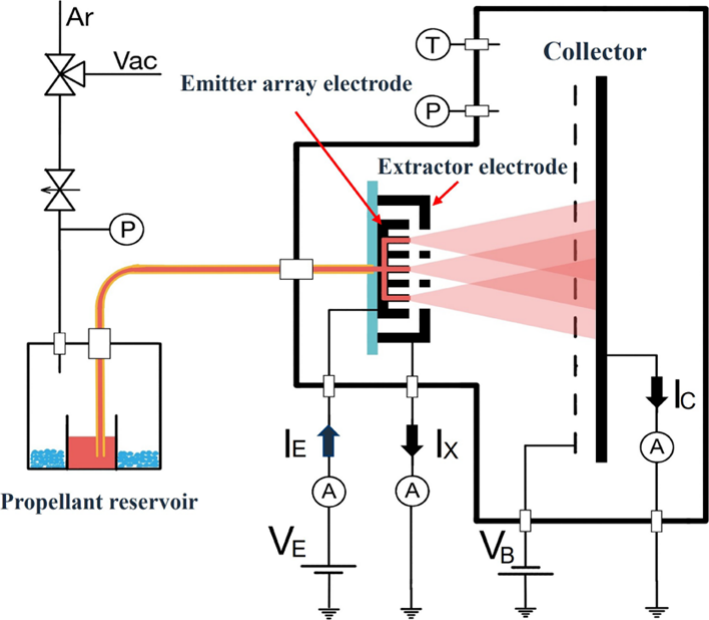
Schematic of the experimental setup.

Figure 9 is a schematic of the experimental setup. The end of the fused silica tube is inserted into a pressure-tight tank and submerged in a vial filled with propellant. For these tests, we electrospray EMI-Im, synthesized by Merck (product number 4.90189). EMI-Im is an ionic liquid often used in electrospray propulsion.^36−38^ It has the very low vapor pressure needed to operate in vacuum and the high electrical conductivity necessary to produce the highly charged droplets and ions of interest in propulsion.

EMI-Im is hydrophilic, and to eliminate potential water absorption, the propellant reservoir is kept under vacuum during 24 h before testing, after which the reservoir is pressurized as needed using argon. As a precaution, the vial sits over a bed of Drierite desiccant for absorbing water vapor molecules that may have entered the system (during testing, we have never observed a change in the color indicating that the desiccant is not dry). Pressurized argon, a Sargent Welch DuoSeal 1405 mechanical pump, an MKS Baratron Type 122A pressure gauge, and a manifold of valves are used to accurately control the pressure *P* in the propellant tank and feed the desired flow rate of EMI-Im to the thruster head. The volumetric flow rate of propellant is given by
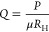
1where μ is the viscosity of EMI-Im. The hydraulic resistance *R*_H_ is the sum of the hydraulic resistances of the fused silica line, the array of conduits in the tubular emitters, and of the network of microchannels
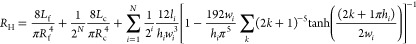
2where *L*_f_ and *R*_f_, and *L*_c_ and *R*_c_ are the length and radius of the fused silica line and the emitters’ conduits, respectively, and *l*_*i*_, *h*_*i*_, and *w*_*i*_ are the length, depth, and width of a microfluidic channel in the *i*th bifurcation. We use 10 000 terms in the series, which leads to a negligible error in the calculation of *R*_H_. The viscosity of EMI-Im exhibits a strong dependence on temperature, which is approximated with the experimental fitting μ = 0.000214 e^692/(*T*–160)^ [kg/m s].^39^ The temperature of the flange holding the thruster head is monitored with a type K thermocouple and a Fluke 116 multimeter. The emitter and extractor electrodes are connected to the output and return of a high voltage power supply, made with an Advanced Energy 10A12-P4 voltage converter, with the return terminal also connected to facility ground. The potential of the emitter is designated by *V*_E_. The currents out of the emitter and into the extractor electrodes, *I*_E_ and *I*_X_, respectively, are measured with shunt resistors. The shunt resistor for the extractor current is placed between the extractor and ground, and the small voltage difference referenced to ground is measured with an LF411 operational amplifier wired in the standard non-inverting amplifier configuration. The shunt resistor for the emitter current is placed in series with the high voltage output, measured with an LF411 operational amplifier in the non-inverting configuration, and this voltage difference is transferred and referenced to ground using a ISO121 isolation amplifier. The beam of charged droplets and ions is intercepted by a large collector electrode, connected to ground through a fast-response electrometer made with an LF411 operational amplifier wired in the standard inverting amplifier configuration. The collector is electrostatically shielded by a screen biased negatively to suppress secondary electron emission. The currents at the emitter, extractor and collector *I*_C_, the pressure, and the temperature are logged with a computer using a National Instruments NI9205 data acquisition card and LabVIEW.

In order to produce the high exhaust velocities needed for propulsion, the electrosprayed droplets must have sufficiently high charge-to-mass ratios, which requires droplet diameters of tens of nanometers or smaller.^13^ These droplets do not scatter enough light to enable the use of optical diagnostics,^40,41^ and vacuum techniques such as retarding potential and time-of-flight are used instead. In particular, the electrosprays of EMI-Im have been thoroughly studied in vacuum to determine the particle composition and the distribution of charge-to-mass ratios,^4,42^ as well as the structure of the beams.^43−45^

We have fabricated and tested four different electrospray sources to characterize the scalability of the thruster head and the microfabrication process, as wells as the sensitivity to geometric parameters such as the distance between the emitters and the extractor. In particular, we have tested a single emitter, two 64 emitter arrays, and a 256 emitter array. Table 1 shows the key characteristics (emitter-extractor gap, microfluidic channel depth, radius and length of fused silica line, the total mass, and the hydraulic resistance) of the thruster heads.

**Table 1:** Design Parameters of the Electrospray Sources Microfabricated and Tested in This Study

	single emitter thruster head	64 emitter thruster head	64 emitter thruster head	265 emitter thruster head
emitter-extractor gap (μm)	280	225	280	350
fused silica line length (cm)	72.0	48.5	96.4	83.4
fused silica line radius (μm)	25	75	125	125
channel depth (μm)	28	27	20	20
dry mass (g)	4.22	4.14	3.98	5.08
total hydraulic resistance (m^–3^)	5.434 × 10^18^	5.433 × 10^16^	3.875 × 10^16^	3.756 × 10^16^

## 4 Results and Discussion

**Figure 10:**
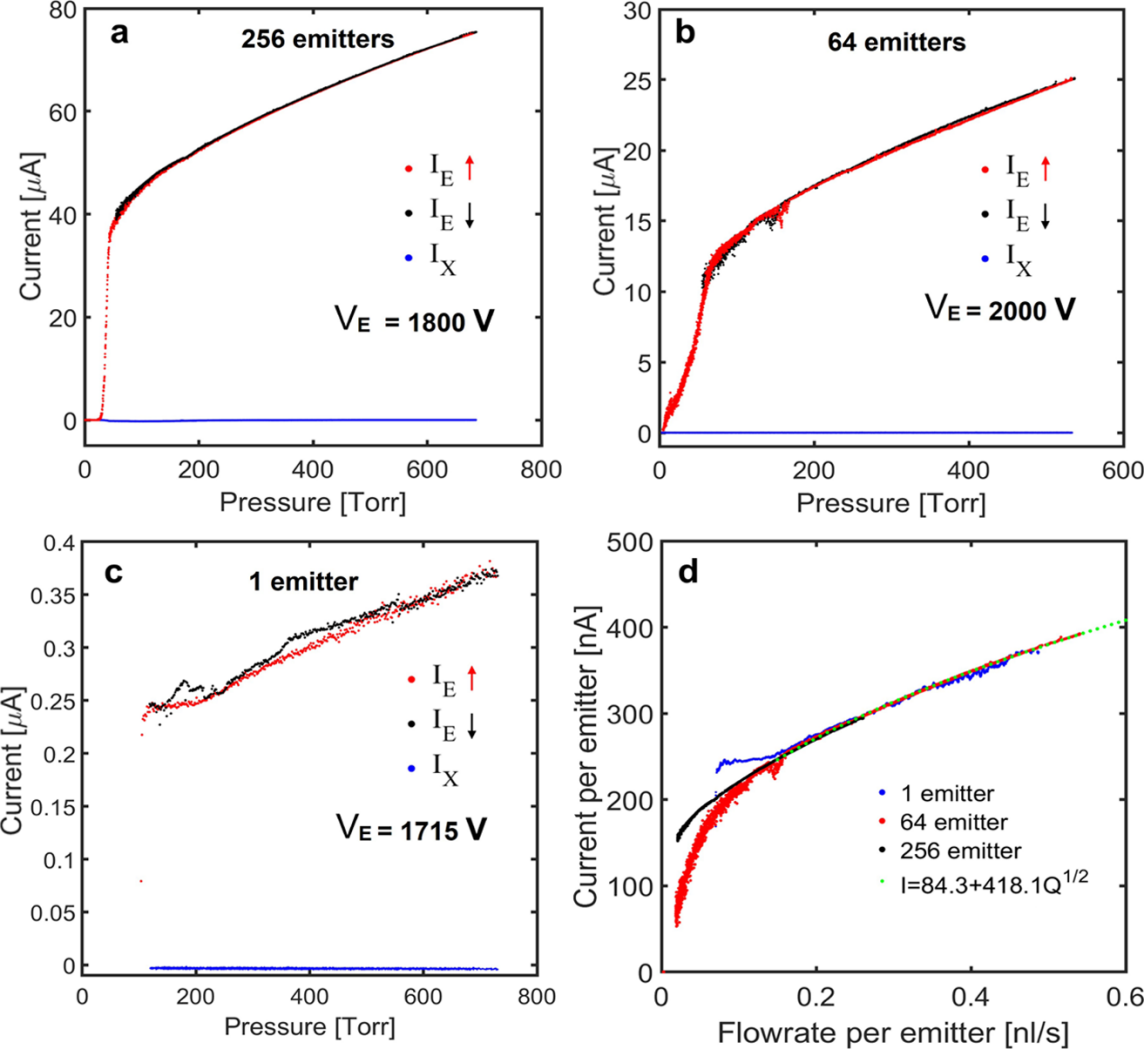
(a) Emitter and extractor currents as a function of pressure for the 256 emitter source; (b) *I*_E_(*P*) and *I*_X_(*P*) for the 64 emitter source; (c) *I*_E_(*P*) and *I*_X_(*P*) for the single emitter source; and (d) current and flow rate per emitter for the three sources.

Figure 10a shows the currents measured at the emitter and the extractor electrodes as functions of the pressure driving the propellant for the 256 emitter source. The pressure is ramped up from *P* = 0 Torr at a fixed emitter potential, *V*_E_ = 1800 V. The current increases sharply in the range 0 < *P* < 55 Torr, as the propellant fills successive rows of emitters. The growth of the *I*_E_(*P*) curve slows down in the 55 Torr < *P* < 120 Torr range, adopting a trend similar to that of normal operation albeit with increased noise. The noise is caused by the intermittent operation of the emitters, which are fed a flow rate of propellant smaller than the minimum required for stable operation.^46^ The emitter current is stable at *P* > 120 Torr, and increases monotonically with pressure. Figure 10a also shows values of the emitter current measured while ramping down the pressure. The two *I*_E_(*P*) curves are identical, indicating the absence of hysteresis in the actuation. This desired feature is made possible by the reduced dead volume and the absence of trapped gas/vapor in the network of microfluidic channels. Note also that the current measured in the extractor is much smaller than the emitter current. While the latter is indicative of thrusting performance, negligible extractor current is key for the lifetime of the thruster because beam interception by the extractor is a main mechanism leading to failure. Figure 10b,c includes the same current characterization for the 64 emitter source and the single emitter source, respectively. The *I*_E_(*P*) trends and the negligible extractor currents are similar for the three sources. Figure 10d compares the current versus flow rate per emitter for the three sources. The three curves are nearly identical above the minimum flow rate, *Q*_min_ ≅ 0.134 nL/s, suggesting that, in each array, all emitters are turned on, and demonstrating the scalability of the array. Although a good match between these curves is expected due to the universality of the *I*_E_(*Q*) characteristic of a cone-jet, the nearly perfect match is partially a coincidence because of the weak dependence of the current on both the voltage difference and the gap between the emitter and the extractor, which are slightly different for the three sources. Note also that the experimental data is well-fitted above the minimum flow rate by *I*_E_ = 418*Q*^1/2^ + 84.3, in agreement with the well-established scaling law *I*_E_ ∝ *Q*^1/2^ for electrosprays operating in the cone-jet mode.^1^ We do not expect the *I*_E_(*Q*) data of the three electrospray sources to coincide below the minimum flow rate because in this case, the emitter current depends on the number of rows in the array, how they are positioned with respect to the vertical direction, and the speed at which the microfluidic channels fill with propellant.

**Figure 11:**
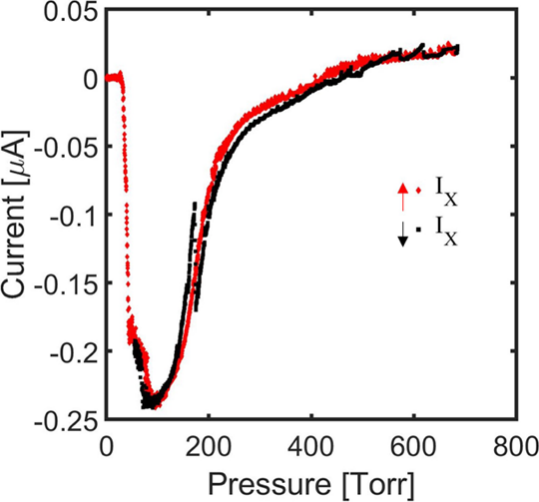
Extractor current as a function of pressure for the 256 emitter source.

Figure 11 reproduces with higher resolution the extractor current of the 256 emitter source. Within this pressure range, the extractor current is always below 0.53% of the beam current. Furthermore, the current is negative in most of the pressure range, indicating that the net flux of charge into the extractor is dominated by negatively charged particles. Because the electrosprayed droplets and ions are positively charged, the current into the extractor is dominated by secondary electrons emitted from the collector grid, which is biased negatively. The emission of secondary electrons from the surfaces of the collector and other electrodes in the vacuum facility is well-known.^47^ Thus, the very small current measured in the extractor is mostly due to facility effects, and it is likely that beam impingement in the extractor is much smaller than the values shown in Figure 11. Note that the extractor current has a minimum at *P* ≅ 30 Torr, it increases rapidly while remaining negative up to *P* ≅ 425 Torr, and then increases more slowly at a higher pressure, reaching very small positive values (0.02% of the beam current at the highest pressure in Figure 11). This suggests that positively charged electrosprayed particles begin to impinge in the extractor as the beamlets become broader at increasing flow rate,^45^ compensating for the current of secondary electrons. The incoming flux of secondary electrons may also be reduced as the beamlets become broader. It is not possible to obtain a more precise description of extractor impingement by the electropray beam in this experimental configuration. A more quantitative characterization will require placing a third electrode downstream of the extractor to shield it from the backflow of secondary electrons.

**Figure 12:**
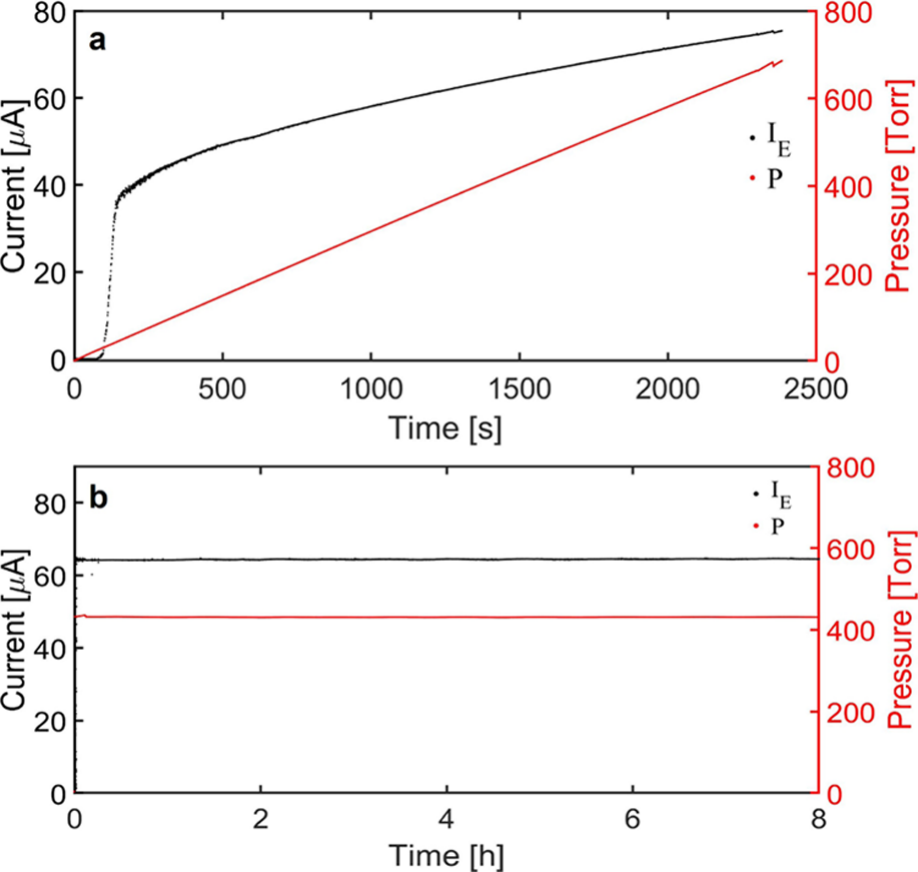
Time response and stability of the emitter current, 256 emitter source: (a) linear pressure ramp and corresponding emitter current; (b) stability of the emitter current when the pressure is kept constant during 8 h.

Figure 12 plots the emitter current and the pressure driving the flow for the 256 emitter source, as functions of time. In Figure 12a, the pressure is ramped up at a constant rate, starting from zero. The pressure and current values are sampled every 0.7 s; the emitter current is stable at frequencies smaller than this sampling rate, except at pressures below the one associated with the minimum flow rate (in this figure *P* ≤ 250 Torr, or equivalently before the 844 s mark). In Figure 12b, the propellant tank is locked for 8 h, keeping the pressure constant except for changes in the room temperature, and the data are sampled at 0.7 s intervals. The average pressure is 430.95 Torr, and its standard deviation is 0.39 Torr. The emitter current, with an average value of 64.41 μA and a standard deviation of 0.10 μA, is remarkably stable within 8 h of operation. Although the current stability of the multi-emitter arrays is encouraging, it will be important to prove that the source does not degrade during tens of days or even a few months of continuous operation, that is, during the expected lifetime of the propulsion system in SmallSat missions.

**Figure 13:**
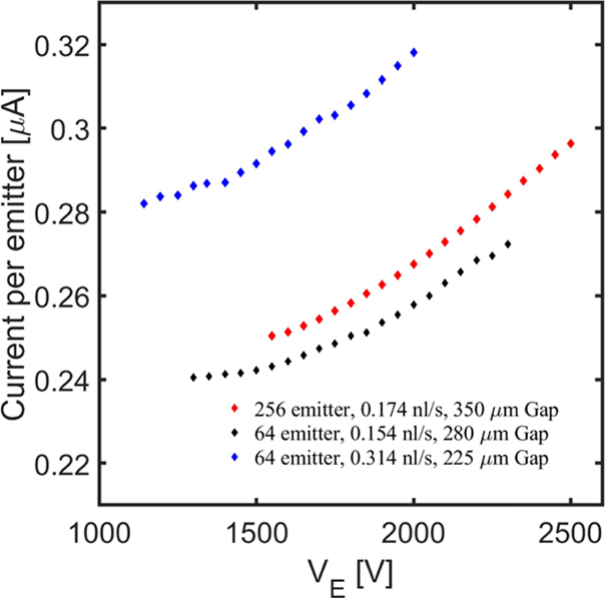
Variation of the current per emitter with emitter voltage for three different electrospray sources.

Figure 13 shows the current per emitter as a function of the emitter voltage for the two 64 emitter sources (they have different gaps between the emitters and the extractor), and for the 256 emitter source. The pressure, and therefore the flow rate of propellant, is fixed in each source. The minimum voltages are representative of the lowest values at which the electrosprays are stable (identified by monitoring the emitter current). The minimum voltages are 1150, 1300, and 1550 V for the emitter-extractor gaps of 225, 280, and 350 μm, respectively. The voltage is increased up to values regarded safe for operation (to prevent shorting between the electrodes), although we suspect that significantly higher voltages can be used. In experiments with macroscopic single emitters at high voltage, imperfections in the axial symmetry of the emitter and extractor electrodes lead to the shifting of the emission point from the axis toward the rim of the emitter, which is accompanied by high extractor impingement. The extractor currents associated with Figure 13 are always negligible, they remain at the levels shown in Figure 11. The lack of beam impingement at the highest emitter voltages is attributed to the excellent alignment observed in Figure 7. Changes in the emitter voltage by 74, 74, and 61% lead to changes in the current by 16, 13, and 18%, where the values are listed at an increasing gap between the electrodes. The variation of the emitter current with the emitter potential is small but not insignificant, and could be used to increase the charge-to-mass ratio of the droplets and therefore the specific impulse of the thruster. We cannot show photographs of the beams and of how they are affected by the potential difference between the emitter and extractor electrodes because the very small droplets of interest to electrospray propulsion do not scatter enough light to make the beams visible. However, the interested reader can find maps of the electric potential and simulations of the structure of the beams for these multi-emitter electrospray sources in ref (48).

**Figure 14:**
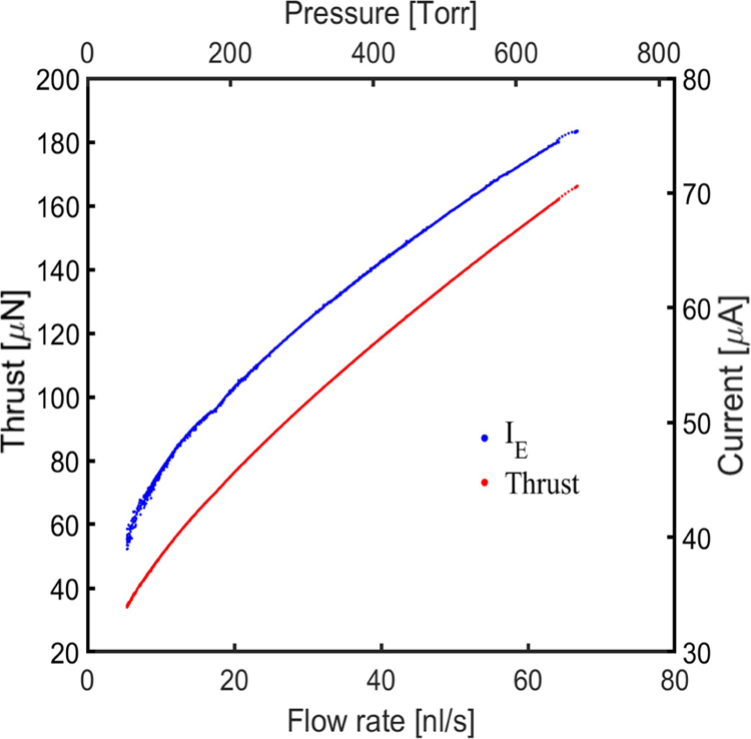
Beam current and estimated thrust for the 256 emitter source, *V*_E_ = 1800 V, as a function of flow rate of propellant.

Figure 14 shows the estimated thrust generated by the 256 emitter source. The thrust is evaluated using the emitter current and the flow rate to compute the average charge-to-mass ratio of the electrosprayed propellant and the exhaust velocity:
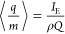
3
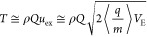
4where ρ is the density of the propellant, ρ = 1519 kg/m^3^ for EMI-Im. Equation 4 overestimates the thrust (e.g., a fraction of the emitter potential is dissipated and not used to accelerate the charged particles, another fraction is converted into the particles’ velocity in the radial direction which does not generate thrust, etc.) but, based on a comparison with a direct thrust measurement to be reported elsewhere, the values shown in Figure 14 are approximately 18% larger than the actual thrust. At the emitter potential, *V*_E_ = 1800 V, and propellant flow rates used in these experiments, the estimated thrust ranges between 34 and 166 μN, that is, between 0.132 and 0.64 μN per emitter. The thrust range depends on the propellant used (which determines  and *Q*) and the emitter potential (which can be increased beyond *V*_E_ by using a third accelerating electrode). For a comparison with similar technologies, electrospray thrusters operating in ion mode and using porous arrays of 480 emitters are reported to deliver between 5 and 22.5 μN, or 0.010 and 0.046 μN per emitter,^32^ while a field emission electric propulsion thruster with and array of 28 emitters is reported to produce a thrust of up to 350 or 12.5 μN per emitter.^49^ A more useful comparison of propulsive performance, including values for the specific impulse, power consumption, thrust to power ratio, propulsive efficiency, total impulse, and total impulse per total mass of the propulsion system, requires the comparison between actual propulsion systems and the use of a thrust stand.

## 5 Conclusions

We have microfabricated and demonstrated a multi-emitter electrospray source for electrospray propulsion. The source has three components (an emitter and an extractor microfabricated in silicon wafers and a glass wafer) anodically bonded into a single element. The emitter electrode features an array of emitters on the topside of the wafer and a matching network of microfluidic channels on the backside. The bonding step includes a process for aligning the emitters and the orifices in the extractor. Furthermore, upon bonding, the glass wafer seals the network of microfluidic channels, providing for a pipe-flow propellant delivery system that evenly distributes the propellant among all emitters. The source is scalable: we have demonstrated arrays of 256, 64, and one emitter microfabricated with the same process; the currents per emitter of the different sources are identical when operated at the same flow rate per emitter; and the microfabrication process can be used to make much larger arrays. The advantages of this design and fabrication process compared to our previous work^34^ are the demonstrated scalability, which is key for applications in SmallSats, and the integration into a single thrusting element with excellent alignment, which is essential for the correct operation, testing, and integration into a propulsion system.

The emitter current follows the well-known *I*_E_ ∝ *Q*^1/2^ scaling law for electrosprays operating in the cone-jet mode. The emitter current does not exhibit hysteresis when ramping up and down the pressure driving the propellant, a desired feature made possible by the small volume of the microfluidic channel network and the absence of trapped gas. The interception of the beam current by the extractor electrode is negligible in the investigated range of flow rates and emitter potentials, which is made possible by the excellent alignment between the emitters and the extractor orifices. Most of the negligible extractor current is due to facility effects (secondary electron emission from collector/grid electrodes), rather than impingement by the electrospray beam. The estimated thrust of the 256 emitter source ranges between 34 and 166 μN in the operational conditions used to characterize it.

Minor improvements to this electrospray source such as the inclusion of a third accelerating electrode will lead to a functional thruster head for electrospray propulsion systems (other standard subsystems such as a power processing unit, a propellant delivery system and a neutralizing cathode are also required). The electrospray source could also be useful for applications requiring fine atomization of liquids at high throughputs such as nanoparticle deposition, electrospinning, and electrospray ionization. Many of these applications are done at atmospheric pressure, and will likely require redesigning the electrodes (e.g., by bringing closer the extractor to the emitters, using a third electrode to apply an electric field downstream of the extractor, etc.) to avoid interception of the spray of droplets or fibers by the extractor and to direct the electrosprayed particles into a collecting area.
